# Pharmacokinetics, safety, and tolerability following single-dose migalastat hydrochloride (GR181413A/AT1001) in healthy male Japanese subjects

**DOI:** 10.3109/21556660.2013.827117

**Published:** 2013-07-24

**Authors:** Hiroko Ino, Naoki Takahashi, Takumi Terao, Paul N. Mudd, Toshiyasu Hirama

**Affiliations:** 1 Medicines Development, Japan Development & Medical Affairs Division, GlaxoSmithKline K.K., TokyoJapan; 2 Biomedical Data Sciences Department, GlaxoSmithKline K.K., TokyoJapan; 3 Clinical Pharmacology, GlaxoSmithKline, Research Triangle Park, NCUSA

**Keywords:** Migalastat HCl, GR181413, AT1001, Pharmacologic chaperone, Fabry disease, Pharmacokinetics, Japanese

## Abstract

**Objective:**

Fabry disease is a rare X-linked disease caused by mutations to the GLA gene, resulting in a deficiency of the lysosomal enzyme alpha-galactosidase A. This study evaluated the pharmacokinetics, safety, and tolerability of ascending single doses of oral migalastat hydrochloride (HCl), an investigational drug, in healthy Japanese volunteers.

**Methods:**

In this phase I, randomized, placebo-controlled, single-blind, ascending single-dose, cross-over study, migalastat HCl (50 mg, 150 mg, or 450 mg) or placebo was administered orally to 14 fasting male Japanese volunteers (aged 20–55 years) on 4 non-consecutive days. Main plasma and urine pharmacokinetic end-points included maximum observed plasma concentration (*C*_max_), time to *C*_max_ (*t*_max_), area under the plasma concentration–time curve (AUC), apparent terminal-phase half-life (*t*_1/2_), urinary recovery of unchanged drug, renal clearance, and percentage of drug excreted in urine. Safety end-points included adverse events, clinical signs and symptoms (e.g., hematology, chemistry, and urinalysis), vital signs (blood pressure and heart rate), and 12-lead electrocardiogram.

**Clinical trial registration number:**

ClinicalTrials.gov registration identifier is NCT01853852.

**Results:**

Median *t*_max_ of migalastat was 3.0–3.5 h. Migalastat HCl concentrations declined relatively rapidly, with a mean *t*_1/2_ of 3.2–4.0 h. The amount of migalastat HCl recovered in the urine and the percentage of migalastat HCl excreted unchanged over 24 h were consistent (∼45–50%) across the dose range. The AUC and *C*_max_ of migalastat HCl were dose proportional from 50–450 mg. Safety results were similar to those observed in non-Japanese populations.

**Conclusions:**

This study demonstrated that ascending single doses of migalastat HCl (50 mg, 150 mg, 450 mg) are absorbed at a moderate rate and eliminated relatively rapidly, with a safety profile consistent with that observed in non-Japanese populations. These results confirm the dose-proportional pharmacokinetics of migalastat HCl from 50–450 mg. This study was limited by a small subject population and a short-term follow-up.

## Introduction

Fabry disease is a rare X-linked disease caused by mutations to the GLA gene that encodes the lysosomal enzyme alpha-galactosidase A (*α*-Gal A)1. Some of the mutations generate mutant proteins that do not fold into the correct 3-dimensional (3-D) shape and, therefore, cannot pass through the endoplasmic reticulum for proper trafficking to the lysosomes. Instead, mutant forms of the enzyme are prematurely degraded, resulting in a deficiency of *α*-Gal A activity2,3.

Under normal conditions, *α*-Gal A is involved in the cellular metabolism of the fatty substance globotriaosylceramide (GL-3). A deficiency of *α*-Gal A activity leads to the accumulation of GL-3 in cells and organs throughout the body and, ultimately, to the life-threatening manifestations of Fabry disease: kidney failure, heart disease, and stroke1,2,4.

The standard of care for Fabry disease is enzyme replacement therapy, which replaces the deficient *α*-Gal A enzyme with regular infusions of recombinant human *α*-Gal A enzyme5. In contrast, a new oral investigative treatment for Fabry disease, called migalastat hydrochloride (migalastat HCl; GR181413A/AT1001/1-deoxygalactonojirimycin HCl; Amicus Therapeutics, Cranbury, NJ, and GlaxoSmithKline, Research Triangle Park, NC), acts as a pharmacologic chaperone by binding to and stabilizing mutant *α*-Gal A, and allowing the mutant forms of the enzyme to adopt an improved 3-D shape. In doing so, migalastat HCl enables passage of mutant *α*-Gal A into the endoplasmic reticulum for proper cellular trafficking to the lysosomes, where it can now operate in a functionally active state within the lysosome, thereby rectifying the enzyme deficiency to a degree6–10.

Phase III trials of patients with Fabry disease, including Japanese participants, are currently under way, investigating oral migalastat HCl monotherapy dosed at 150 mg every other day (ClinicalTrials.gov identifiers NCT00925301 and NCT01218659). This phase I study (ClinicalTrials.gov identifier: NCT01853852) was the first investigation of migalastat HCl to be conducted in Japanese subjects. The objective was to evaluate the pharmacokinetics, safety, and tolerability of ascending single doses of oral migalastat HCl in healthy Japanese volunteers.

## Patients and methods

### Study design

This was a randomized, placebo-controlled, single-blind, ascending single-dose, cross-over study in healthy Japanese volunteers. Migalastat HCl or placebo was administered orally to volunteers on 4 different days separated by at least 7 days. As shown in the dosing schedule (Table 1), on 3 of the 4 dosing days, volunteers received a 50-mg, 150-mg, or 450-mg dose in ascending order in the fasted state. Volunteers were randomly allocated to one of four dosing sequences differentiated by what day placebo was given. This study was conducted in line with the 2008 Declaration of Helsinki, Good Clinical Practice principles, and applicable regulatory requirements at the GlaxoSmithKline Medicines Research Unit in Prince of Wales Hospital, Randwick, Australia. Informed consent was obtained from the participants prior to study start. The study protocol and relevant documents were reviewed and approved by the Bellbery Human Research Ethics Committee in Dulwich, South Australia.

**Table 1.** Planned subject number and dosing schedules.

Sequence	*n*	Dose 1	Dose 2	Dose 3	Dose 4
A	3	50 mg migalastat HCl	150 mg migalastat HCl	450 mg migalastat HCl	Placebo
B	3	50 mg migalastat HCl	150 mg migalastat HCl	Placebo	450 mg migalastat HCl
C	3	50 mg migalastat HCl	Placebo	150 mg migalastat HCl	450 mg migalastat HCl
D	3	Placebo	50 mg migalastat HCl	150 mg migalastat HCl	450 mg migalastat HCl

HCl, hydrochloride.

### Subjects

This trial enrolled healthy Japanese volunteers. ‘Japanese’ was defined as being born in Japan, having four ethnic Japanese grandparents, holding a Japanese passport or identity papers, and having the ability to speak Japanese, in addition to not living outside of Japan for more than 10 years. Criteria for inclusion were male or female sex, aged between 20–55 years, body weight ≥50 kg, and body mass index between 18.5–29.0 kg/m^2^. Women of non-childbearing potential were eligible. Exclusion criteria included positive pre-study results within 3 months of screening for the following conditions: hepatitis B, hepatitis C, or human immunodeficiency virus; positive pre-study results for an alcohol/drug screen; regular use of alcohol and/or tobacco within 6 months of study start; exposure to four new chemical entities within 1 year prior to first study dose; and use of an investigational product in a clinical trial, or use of prescription and non-prescription drugs, within 7 days or 5 half-lives of the first study dose. Women who were pregnant or lactating were ineligible. Participants were permitted to take paracetamol (≤2 g daily).

### Procedures

Blood samples of 2 mL for measuring plasma concentrations of migalastat were collected into a tube containing the anticoagulant EDTA-3K prior to dosing and at 0.5, 1, 1.5, 2, 2.5, 3, 3.5, 4, 5, 6, 8, 10, 12, 16, and 24 h post-dose. Blood samples were mixed thoroughly and kept on crushed ice until the samples were centrifuged at ∼1500 × *g* for 15 min to obtain plasma. Plasma samples were prepared within 1 h of collection and stored at −20°C until analysis.

Urine samples were collected prior to dosing and at 0–4 h, 4–8 h, 8–12 h, and 12–24 h after dosing. Urine samples were refrigerated at 2–8°C immediately after collection, divided into 1-mL aliquots, and stored at −20°C until analysis.

Concentrations of migalastat in plasma and urine were determined using validated liquid chromatography-tandem mass spectroscopy methods developed by Celerion (Lincoln, NE). The analytical ranges for migalastat were 5.88–2940 ng/mL in plasma and 100–5000 ng/mL in urine.

### Study drug

**Figure 1.**
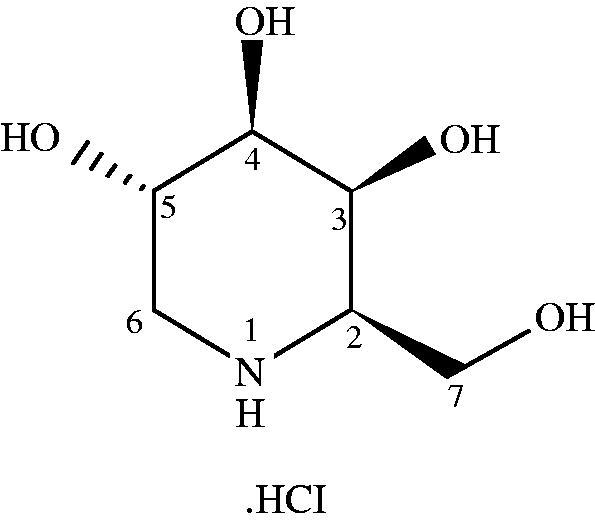
Chemical structure of migalastat hydrochloride (GR181413A/AT1001)/1-deoxygalactonojirimycin hydrochloride).

Migalastat HCl is a low molecular weight iminosugar, an analog of the terminal galactose group that is cleaved from the substrate GL-3 (Figure 1). Migalastat HCl is being codeveloped by Amicus Therapeutics (Cranbury, NJ) and GlaxoSmithKline (Research Triangle Park, NC). The dosage forms used in this phase I trial were capsules (150 mg and 450 mg) and oral solution (50 mg). Matching placebo was used in capsule form and oral solution.

### Pharmacokinetic parameter assessments

Each pharmacokinetic parameter for migalastat was calculated by non-compartmental methods using version 4.1 of the software program WinNonlin (Pharsight, St Louis, MO). Plasma concentration–time data for migalastat were used to calculate the following parameters: maximum observed plasma concentration (*C*_max_), time to *C*_max_ (*t*_max_), last time of quantifiable concentration, terminal-phase rate constant, area under the plasma concentration–time curve [AUC_(0–_*_t_*_)_ and AUC_(0–∞)_], percentage of AUC_(0–∞)_ obtained by extrapolation, apparent terminal-phase half-life (*t*_1/2_), apparent clearance following oral dosing, and apparent volume of distribution based on terminal phase. The urine concentration–time data of migalastat were used to calculate urinary recovery of unchanged drug, renal clearance, and percentage of the drug excreted in urine.

### Safety and tolerability assessments

Participants underwent the following assessments at screening, the day before and after each dose, and at the follow-up visit (7–10 days after the last dose): vital signs (diastolic and systolic blood pressure, heart rate, and body temperature); clinical laboratory tests (i.e., clinical chemistry, hematology, and urinalysis); adverse events (AEs) and serious AEs (SAEs); and 12-lead electrocardiogram (ECG). Subjects received a complete physical examination up to 28 days prior to study start, and a brief physical examination 2 hours prior to dosing and at the follow-up visit.

### Statistical analyses

Summary statistics (*n*, mean [95% confidence interval (CI)], standard deviation [SD], minimum, median, maximum, geometric mean [95% CI], SD on log-scale, and between-subject coefficient of variation [CV]) were derived for each pharmacokinetic parameter per dose. Derived plasma and urine pharmacokinetic parameters were plotted graphically for each dose to demonstrate the pharmacokinetic–dose relationship. Dose proportionality for pharmacokinetic parameters of migalastat was analyzed using AUC and *C*_max_, and the following power model with 90% CI: log (pharmacokinetic parameter) = μ + *S_i_* + *P_j_* + β*log(*D_k_*) + *ε_ijk_*, where μ is the overall mean, *S_i_* is the random effect for subject *i* following normal distribution *N*(0, 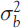), *P_j_* is the fixed period effect, *β* is the slope of log_e_-transformed dose, *D_k_* is the dose, and *ε_ijk_* is the random error following normal distribution *N*(0, 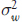).

## Results

### Subject characteristics and demographics

Fourteen volunteers were enrolled and 13 completed the study (Table 2). One subject withdrew consent because of personal reasons, after receiving the 50-mg dose. All the volunteers were Japanese males, with a mean (SD) age of 28 (5) years (range = 22–38 years) and a mean (SD) body weight of 64 (8) kg (range = 54–89 kg).

**Table 2.** Subject demographics and baseline characteristics.

Parameter	*n*	Mean (SD)	Median	Range
Sex				
Male	14	NA	NA	NA
Female	0	NA	NA	NA
Age (years)	14	28.4 (4.89)	27.5	22–38
Height (cm)	14	170.1 (5.08)	170.0	163–178
Weight (kg)	14	63.54 (8.456)	61.05	53.9–89.0
BMI (kg/m^2^)	14	21.96 (2.702)	21.10	19.4–28.4

BMI, body mass index; NA, not applicable; SD, standard deviation.

### Single-dose pharmacokinetics of migalastat HCl

**Figure 2.**
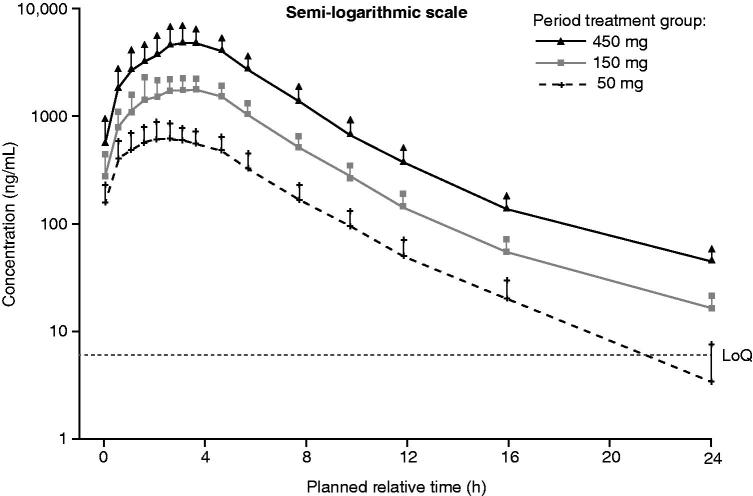
Mean (standard deviation) plasma concentration-time profiles of migalastat hydrochloride after single oral 50-, 150-, and 450-mg doses in healthy male Japanese volunteers in the fasted state. LoQ, limit of quantification.

Figure 2 presents the mean plasma concentration–time profiles of migalastat in the fasting state. The mean pharmacokinetic parameters of migalastat are summarized in Table 3. Migalastat HCl (50 mg, 150 mg, and 450 mg) was absorbed at a moderate rate following ascending single-dose oral administration, reaching median *t*_max_ within 3.0–3.5 h post-dose in the fasted state. These concentrations then declined relatively rapidly, with a mean *t*_1/2_ of between 3.2–4.0 h. Migalastat clearance (∼13–15 L/h) and the percentage of migalastat HCl excreted in urine unchanged over 24 h (∼45–50%) were generally consistent across the dose range of 50–450 mg and, therefore, suggest dose proportionality (Table 3).

**Table 3.** Summarized pharmacokinetic parameters of migalastat hydrochloride following single oral 50-, 150-, and 450-mg doses in healthy male Japanese volunteers in the fasted state.

Dose	*n*	Pharmacokinetic parameter								
		*C*_max_ (ng/mL)	*t*_max_* (h)	AUC_(0–_*_t_*_)_ (h·ng/mL)	AUC_(0–∞)_ (h·ng/mL)	*t*_1/2_ (h)	CL/F (L/h)	Ae_(0–24)_ (mg)	CLr (L/h)	%FX_(0–24)_ (%)
50 mg	14	695.1 (36)	3 (1.5–5)	3,905.4 (35)	3,961.5 (34.6)	3.176 (22.5)	12.621 (34.6)	24.84 (24.6)	6.303 (20.6)	49.67 (24.6)
150 mg	13	2124.3 (36.3)	3.5 (2–5)	11,430.7 (27.4)	11,519.4 (27.4)	3.816 (6.6)	13.022 (27.4)	69.97 (22.9)	6.121 (17.8)	46.65 (22.9)
450 mg	13	5694.9 (40.4)	3.5 (2.5–5)	30,453.9 (34.2)	30,721.7 (34.1)	4.009 (5.6)	14.648 (34.1)	200.43 (20.1)	6.008 (16)	44.54 (20.1)

Geometric mean presented with between-subject coefficient of variation. *Arithmetic median (range). %Fx_(0–24)_, percentage of the drug excreted in urine over 24 h; Ae_(0–24)_, urinary recovery of unchanged drug over 24 h; AUC_(0–∞)_, area under the concentration–time curve from time zero (pre-dose) extrapolated to infinite time; AUC_(0–_*_t_*_)_, area under the concentration–time curve from time zero (pre-dose) to last time of quantifiable concentration; CL/F, apparent clearance following oral dosing; CLr, renal clearance; *C*_max_, maximum observed plasma concentration; *t*_1/2_, apparent terminal-phase half-life; *t*_max_, time to *C*_max_.

**Figure 3.**
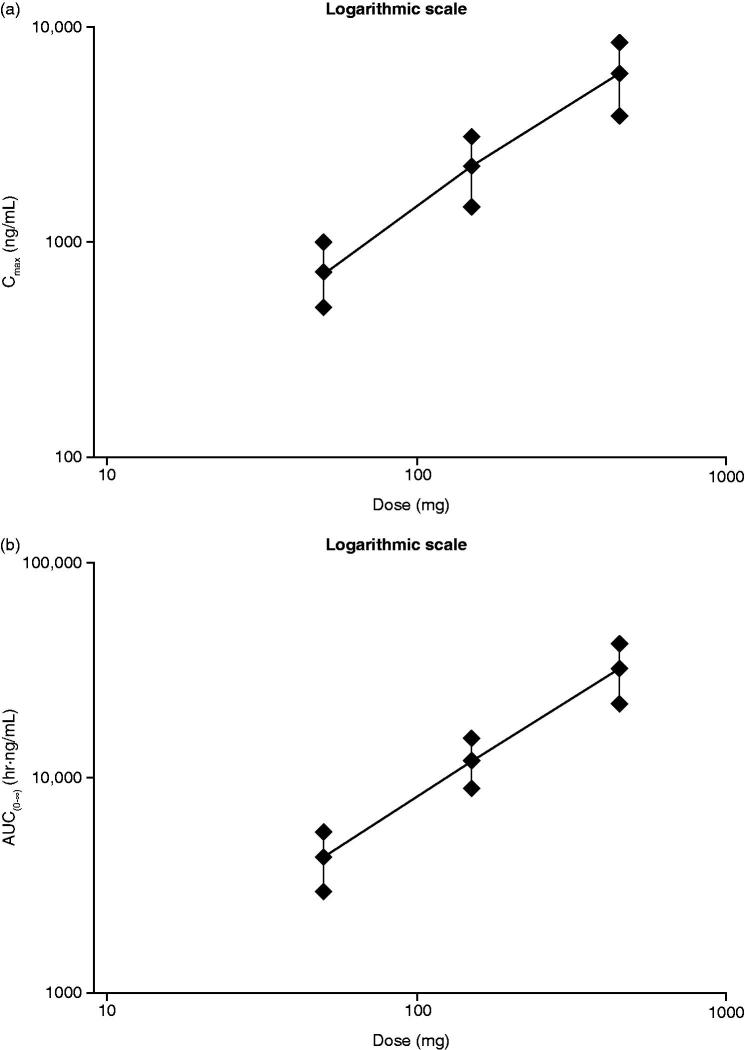
Dose proportionality of (a) *C*_max_ and (b) AUC_(0–∞)_ following single oral 50-, 150-, and 450-mg doses of migalastat HCl in the fasted state. AUC_(0–∞)_, area under the concentration--time curve from time zero (predose) extrapolated to infinite time; *C*_max_, maximum observed plasma concentration; HCl, hydrochloride; SD, standard deviation.

The relationship between a dose of migalastat HCl (50 mg, 150 mg, and 450 mg) and *C*_max_ and AUC is shown in Figure 3, with consistent increases in each pharmacokinetic parameter suggesting a dose-proportional association. The confidence intervals around slope contained 1 for each parameter, again suggesting dose-proportionality: *C*_max_, 0.95 (90% CI = 0.77, 1.14); AUC_(0–_*_t_*_)_, 1.09 (90% CI = 0.92, 1.27); and AUC_(0–∞)_, 1.09 (90% CI = 0.91, 1.26).

### Safety and tolerability

No deaths, SAEs, or withdrawals due to AEs were reported as a result of treatment with migalastat HCl. The most common AE was headache, occurring after dosing with placebo (*n = *2), migalastat HCl 50 mg (*n = *4), and migalastat HCl 450 mg (*n = *2). Treatment-related AEs were reported by six (43%) subjects (Table 4). No correlation emerged between the number or severity of treatment-related AEs and concentrations of migalastat. No migalastat HCl-related clinically significant changes in laboratory values, vital signs, ECG, or physical examination were reported.

**Table 4.** Summary of treatment-related adverse events at each dose level.

	Regimen				
Adverse event	Placebo (*n = *14)	50 mg (*n = *14)	150 mg (*n = *13)	450 mg (*n = *13)	Total* (*n = *14)
Any AE	1 (7)	4 (29)	0	2 (15)	6 (43)
Headache	0	3 (21)	0	2 (15)	5 (36)
Frequent bowel movements	1 (7)	0	0	0	1 (7)
Seasonal allergy	0	1 (7)	0	0	1 (7)
Blood creatinine phosphokinase increased	0	1 (7)	0	0	1 (7)
Arthralgia	0	1 (7)	0	0	1 (7)
Hypertension	0	1 (7)	0	0	1 (7)

Data presented as *n* (%). *A total number of subjects experiencing AEs from period 1 to period 4. AE, adverse event.

## Discussion

This first-in-Japanese, phase I, randomized, placebo-controlled, cross-over study assessed the pharmacokinetics, safety, and tolerability of ascending single doses of migalastat HCl (50 mg, 150 mg, and 450 mg) in the fasted state. Migalastat exhibited a moderate absorption rate, relatively rapid elimination, and dose-proportional pharmacokinetics. The safety profile seen in this study of healthy Japanese males was consistent with the known safety profile of that in non-Japanese populations11. Although the pharmacokinetic data presented in this study are precedented by data in non-Japanese subjects, the data are integral to defining the optimal regimen for migalastat HCl in Japanese patients with Fabry disease.

As with any pharmacokinetics study, potential limitations of this study include the small subject population and the short-term follow-up period. The follow-up period of 7–10 days after receipt of the final dose was long enough to characterize the pharmacokinetics of migalastat HCl but might not be sufficient time to detect AEs unrelated to pharmacokinetics. The potential emergence of such AEs should be examined in clinical studies in subjects over a longer treatment period.

This study corroborates the pharmacokinetic and tolerability findings for migalastat HCl reported in other studies of healthy non-Japanese volunteers11. In three recent ascending single-dose, phase I, randomized, placebo-controlled clinical trials (FAB-CL-101, FAB-CL-104, and AT1001-010), migalastat HCl (capsules or oral solution) was evaluated in 108 fasting, healthy male and female volunteers (age range = 21–55 years) in the US11. Doses in these studies ranged from 25–2000 mg. Pharmacokinetic findings were similar overall for doses of migalastat HCl ranging from 75–1250 mg. For example, the mean *C*_max_ and AUC_0–∞_ values increased in a dose-proportional manner for doses ranging from 75–1250 mg. However, at the higher doses of 1250 mg and 2000 mg, mean *C*_max_ and AUC_0–∞_ were similar and did not increase in a dose-proportional manner. Mean AUC_0–_*_t_* and *C*_max_ values generally increased in a dose-proportional manner between 75–675 mg. However, AUC values increased more than dose proportionally between 25–75 mg, likely due to the fact that the terminal elimination profile was not easily assessed at the lower 25-mg dose. Mean *C*_max_ values generally increased proportionally over the 25–675-mg dose range.

The similarities in the pharmacokinetics of migalastat HCl in Japanese and non-Japanese populations were particularly evident for the 150-mg dose, which is the dose being evaluated in phase III trials11. For example, in study AT1001-010 of healthy non-Japanese volunteers (*n = *51), after single doses of migalastat HCl 150 mg, the geometric mean (CV) values of AUC_0–∞_ and *C*_max_ were comparable with those of the healthy Japanese volunteers in this study (AUC_0–∞_ = 10,449 [25] ng·h/mL vs 11,519 [27] ng·h/mL; *C*_max_ = 1635 [27] ng/mL vs 2124 [36] ng/mL, respectively).

## Conclusion

This phase I study demonstrated that ascending single doses of migalastat HCl (50 mg, 150 mg, and 450 mg), given to healthy Japanese males in the fasted state, are absorbed at a moderate rate, are relatively rapidly eliminated, and demonstrate a safety profile consistent with that of non-Japanese populations. The study also confirmed the dose-proportional pharmacokinetics of migalastat HCl from 50–450 mg.

## Transparency

### Declaration of funding

This study and its publication were funded by GlaxoSmithKline, Research Triangle Park, NC, and Amicus Therapeutics, Cranbury, NJ.

### Declaration of financial/other relationships

PNM Jr is employed by GlaxoSmithKline, USA, and holds stock in the company. HI, NT, TT, and TH are employed by GlaxoSmithKline K.K., Japan. TH holds stock in the company. None of the other Japanese authors hold stocks in the company.

## Acknowledgments

The authors would like to thank Jennifer Yuan, MD, PhD, and Paul S. Thomas, MD, PhD (study investigators), and study volunteers for their participation, as well as Franziska Loehrer, PhD, MRQA, Cheryl Friend, and the staff at GlaxoSmithKline Medicines Research Unit in Sydney, Australia, for support in conducting the study. Professional medical writing and editorial assistance was provided by Stephanie Finucane, MS, CMPP of Caudex Medical, New York, and was funded by Amicus Therapeutics and GlaxoSmithKline.
